# Cornuside mitigates acute lung injury through suppression of NLRP3 inflammasome-mediated pyroptosis and activation of the Keap1-Nrf2 antioxidant response

**DOI:** 10.3389/fphar.2025.1682523

**Published:** 2025-10-08

**Authors:** Tong Luo, Ai Liya, Feng Wang, Wei Yan, Saiyin Chaoketu

**Affiliations:** ^**1**^ Department of Sports Rehabilitation, School of Physical Education and National Equestrian Academy, Wuhan Business University, Wuhan, China; ^**2**^ School of Life Sciences, Beijing University of Chinese Medicine, Beijing, China; ^**3**^ Research Center for Modern Equine Industry Development, Wuhan, China; ^**4**^ Department of Wu-Liao and Rehabilitation, International Mongolian Hospital of Inner Mongolia, Hohhot, China; ^**5**^ Graduate School, Inner Mongolia Medical University, Hohhot, China

**Keywords:** cornuside, acute lung injury, inflammation, pyroptosis, NLRP3 inflammasome, Keap1-Nrf2 pathway

## Abstract

**Introduction:**

Acute lung injury (ALI) is a life-threatening respiratory disorder characterized by excessive inflammation and oxidative stress, with no specific pharmacological therapy currently available. Cornuside (CNS), a bioactive iridoid glycoside derived from *Cornus officinalis* (Sieb. et Zucc.), has garnered increasing attention for its bone-protective, neuroprotective, anti-inflammatory, and anti-diabetic properties, yet its effects on ALI remain unclear.

**Methods:**

Male C57BL/6J mice received intratracheal lipopolysaccharide to induce ALI and intragastric CNS (25 or 50 mg/kg) 1 h before and 3 h after LPS. Lung injury was assessed by survival, wet/dry ratio, bronchoalveolar lavage fluid (BALF) protein, histology, and open-field testing. Oxidative stress was evaluated by MPO, MDA, and GSH-PX assays. Keap1-Nrf2 pathway activation was analyzed by Western blot and immunofluorescence of Keap1, Nrf2, GPX4, and NQO1, including Nrf2 nuclear translocation. *In vitro*, bone-marrow-derived macrophages and J774A.1 cells were used to measure NLRP3 inflammasome activation, caspase-1 cleavage, IL-1β release, and GSDMD-mediated pyroptosis by ELISA, Western blot, confocal imaging, and propidium iodide staining. Lung RNA sequencing identified differentially expressed genes and enriched pathways related to oxidative stress and inflammation.

**Results:**

CNS significantly improved survival, reduced pulmonary edema, and alleviated lung inflammation and locomotor deficits in LPS-challenged mice. Transcriptomic analysis revealed downregulation of oxidative stress- and inflammation-related pathways. CNS inhibited NLRP3 inflammasome activation, as shown by decreased caspase-1 cleavage, IL-1β release, GSDMD processing, and ASC speck formation *in vivo* and *in vitro*. In parallel, CNS activated the Keap1-Nrf2 pathway, increasing nuclear Nrf2 translocation and the expression of antioxidant proteins (GPX4, NQO1), while reducing oxidative stress markers MPO and MDA.

**Discussion:**

These findings demonstrate that CNS protects against LPS-induced ALI by concurrently suppressing NLRP3 inflammasome-mediated pyroptosis and enhancing Keap1-Nrf2 antioxidant signaling. This dual mechanism highlights CNS as a promising natural therapeutic candidate for ALI and related oxidative stress-driven lung diseases.

## Graphical Abstract

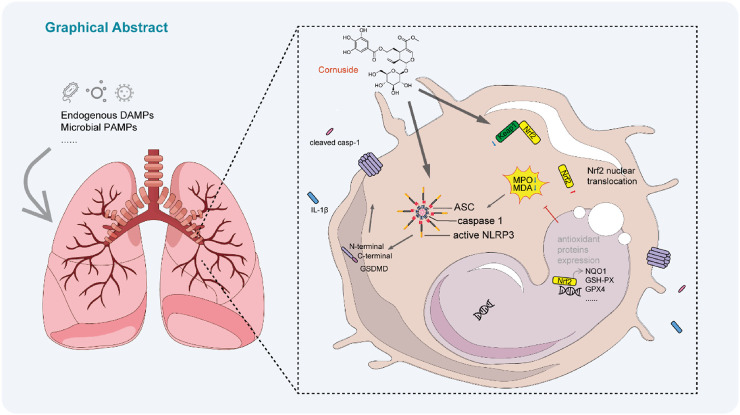

## Introduction

Acute lung injury (ALI) is a common, life-threatening clinical syndrome that has recently attracted considerable global attention, particularly in the context of the coronavirus disease 2019 (COVID-19) pandemic (Long et al., 2022). Pneumonia-the leading cause of ALI-is a lower respiratory tract infection of the lung parenchyma, most often caused by respiratory viruses, Gram-negative or Gram-positive bacteria, and, globally, mycobacteria (Aliberti et al., 2021). Clinically, ALI is characterized by pulmonary edema, infiltration of inflammatory cells, increased microvascular permeability, and, in severe cases, progression to acute respiratory distress syndrome (ARDS) (Xia et al., 2022; Fan et al., 2018). Although advances such as mechanical ventilation have improved patient outcomes, there remains no effective pharmacological treatments for ALI/ARDS, and the mortality rate continues to be unacceptably high, ranging from 35% to 55% (Pham and Rubenfeld, 2017).

The NLRP3 inflammasome is a key component of the innate immune system’s first line of defense against pathogenic infections. Although appropriate activation of NLRP3 helps clear pathogens and supports tissue repair, persistent or excessive activation drives overproduction of downstream cytokines beyond normal host-protective levels (Wang X. et al., 2023). Such dysregulation disrupts the protective balance and contributes to the pathogenesis of ALI and other inflammatory diseases, making NLRP3 inhibition an attractive therapeutic strategy (Yin et al., 2023). Structurally, the NLRP3 inflammasome is a cytoplasmic multiprotein complex composed of the sensor NLRP3, the adaptor ASC (apoptosis-associated speck-like protein containing a CARD), and the effector protease caspase-1. Upon activation, caspase-1 cleaves pro-IL-1β and gasdermin D (GSDMD), generating mature IL-1β and the GSDMD-N fragment. This process induces pyroptosis-a pro-inflammatory form of programmed cell death-and drives the release of inflammatory cytokines, thereby amplifying the inflammatory response (Ren et al., 2024). Consistent with this mechanism, elevated levels of IL-1β and IL-18 are frequently detected in patients with ALI and correlate with poor clinical outcomes (Dolinay et al., 2012). Supporting these clinical observations, multiple experimental models-including LPS-induced septic ALI and ventilator-induced lung injury-have shown that stimuli such as mitochondrial DNA, bacterial endotoxin, and mechanical stress activate NLRP3 through Toll-like receptor (TLR4/TLR9), NF-κB, and p38 MAPK signaling pathways, whereas genetic deletion or pharmacological inhibition of NLRP3 markedly reduces lung inflammation and tissue damage (Wu et al., 2019; Yang J et al., 2022; Zhang et al., 2020; Kuipers et al., 2012; Dai et al., 2015).

In the lung, excessive ROS are generated by inflammatory cells such as macrophages and neutrophils through enzymes including NADPH oxidase and myeloperoxidase (MPO) (Jiang et al., 2020). High ROS levels damage lipids, proteins, and DNA, disrupt the alveolar-capillary barrier, and promote pulmonary edema and inflammatory cell infiltration-hallmark features of ALI (Wang et al., 2025). Clinical and experimental studies consistently show that antioxidant therapies or genetic enhancement of antioxidant pathways can attenuate lung injury and improve outcomes (Zhang Y. et al., 2022; Qin et al., 2024). Importantly, oxidative stress also serves as a major upstream signal for activation of the NLRP3 inflammasome, linking redox imbalance to the amplification of inflammatory cascades in ALI (Zhou et al., 2011; Meng et al., 2023). Conversely, activation of the Kelch-like ECH-associated protein 1 (Keap1)-nuclear factor erythroid 2-related factor 2 (Nrf2) antioxidant pathway mitigates oxidative stress and suppresses NLRP3 activation. Under oxidative conditions, Nrf2 is released from Keap1 inhibition, translocates into the nucleus, and induces the expression of antioxidant and cytoprotective genes, thereby establishing an anti-inflammatory environment that counteracts NLRP3-driven inflammation (Zhao et al., 2021; Tao et al., 2021).

*Cornus officinalis* (Sieb. et Zucc.), a traditional East Asian medicinal herb, has long been prescribed for conditions such as hypertension, lower back and knee pain, dizziness, tinnitus, impotence, nocturnal emissions, and excessive menstruation (Spychaj et al., 2021). During the COVID-19 pandemic, *Cornus officinalis* has also played an important therapeutic role. Since 2020, its use has been recommended in China’s updated Diagnosis and Treatment Protocols for COVID-19, particularly for the management of severe cases that present with ALI or ARDS (Liu et al., 2025). Pharmacological studies have revealed that the high-polarity components of *C*. *officinalis*, especially cornuside (CNS, PubChem CID: 11228694), are responsible for many of its biological activities (Wang et al., 2018). CNS, an iridoid glycoside isolated from the fruit or stone of *C*. *officinalis*, exhibits diverse therapeutic properties, including bone-protective, neuroprotective, anti-inflammatory, and anti-diabetic effects, and has attracted increasing attention in recent years (Gao et al., 2021; Wang L. et al., 2023; Shi et al., 2022; Yu et al., 2023; Lian et al., 2024; Xiang et al., 2024; Cui et al., 2024). However, despite these promising pharmacological activities, the potential role of CNS in treating ALI, as well as its underlying molecular mechanisms, remains largely unexplored.

In this study, we evaluated the protective effects of CNS on lipopolysaccharide (LPS)-induced ALI and demonstrated that CNS markedly reduced lung inflammation and improved locomotor activity in mice. Mechanistically, CNS suppressed NLRP3 inflammasome activation, limited pyroptosis, and enhanced the Keap1-Nrf2 antioxidant pathway, indicating its potential as a promising therapeutic candidate for ALI.

## Materials and methods

### Reagents and antibodies

ATP salt hydrate (A6419), lipopolysaccharides (L4391, L2880), Hoechst33342 (B2261), and propidium iodide (P4170) were purchased from Sigma-Aldrich (Munich, Germany). Nigericin (TLRL-NIG) was from InvivoGen (San Diego, CA, USA), and cornuside (HY-N0631; C_24_H_30_O_14_; Mw: 542.49; purity >99.99%) was purchased from MedChemExpress (Monmouth Junction, NJ, USA). Cell Counting Kit-8 (CK04-100) were obtained from Dojindo (Kumamoto, Japan). Mouse IL-1β enzyme-linked immunosorbent assay (ELISA) kits (KE10003-96T) were purchased from Proteintech (Chicago, IL, USA). Fetal bovine serum (1600044) and Dulbecco’s modified Eagle’s medium (DMEM; C11995500BT) were purchased from Gibco (Carlsbad, CA, USA). Murine M-CSF (315-02) was obtained from PeproTech (Rocky Hill, NJ, USA). Anti-Nrf2 (1:1000, GB113808), anti-IL-1β (1:1000, GB11113-100), anti-GPX4 (1:2000, GB124327), anti-NQO1 (1:1000, GB11282), MDA kits (G4300-96T) and anti-Keap1 (1:1000, GB113747-100) antibodies were obtained from Servicebio (Wuhan, China). GSH-PX kits (A005) were purchased from Jiancheng Bioengineering Institute (Nanjing, China). Anti-GSDMD (1:1000, ab209845), anti-caspase-1 (1:2000, ab179515), and caspase1 P10 Polyclonal antibodies (PA5-105049) were purchased from Abcam (Cambridge, MA, USA). Anti-NLRP3 (1:1000, AG-20B-0014-C100) and Anti-ASC (1:1000, AG-25B-0006-C100) antibodies were obtained from Adipogen (San Diego, CA, USA).

### Mice

Male C57BL/6J mice (8 weeks old) were obtained from Vital River Co., Ltd. (Beijing, China) and acclimated for 1 week in a specific pathogen-free (SPF) animal facility before the start of the experiments (Approval No. BUCM-2024030701-1136). All animal procedures were performed in accordance with the Guidelines for the Care and Use of Laboratory Animals issued by the Ministry of Science and Technology of China and were approved by the Animal Ethics Committee of Beijing University of Chinese Medicine. Mice were randomly assigned to four groups: control, LPS model, CNS low-dose (25 mg/kg), and CNS high-dose (50 mg/kg), Sample sizes (n) for each experiment are indicated in the corresponding figure legends.

### LPS-induced ALI model

Mice were anesthetized with isoflurane, and ALI was induced by intratracheal instillation of LPS at a dose of 2 mg/kg or 10 mg/kg (lethal dose). CNS (25 or 50 mg/kg) or phosphate-buffered saline (PBS) was administered intragastrically 1 h before and 3 h after LPS challenge (Wei et al., 2024). Control mice received equal volumes of PBS intragastrically and intratracheally on the same schedule but without LPS or CNS. The CNS doses were selected from published *in vivo* studies demonstrating efficacy and safety within this range (Wang L. et al., 2023; Yu et al., 2023). The LPS doses were chosen according to established models and confirmed by our preliminary experiments (Nguyen et al., 2022; Shan et al., 2024). Six hours after LPS administration, mice were euthanized with pentobarbital. BALF was collected from a dedicated cohort by instilling 1 mL of cold PBS (4 °C) into the trachea and withdrawing the fluid three times (total 3 mL). The pooled lavage was centrifuged at 300 × g for 5 min at 4 °C to remove cells/debris and the supernatant was used exclusively for BALF assays. These animals were not used for histology or Western blot. Lung tissues for H&E, IHC, IF, RNA-seq, and Western blot were obtained from a separate, non-lavaged cohort; lungs were fixed in 4% paraformaldehyde for histology or snap-frozen in liquid nitrogen and stored at −80 °C for subsequent analysis.

### Open field test

The open field test was performed 6 h after LPS-induced ALI to evaluate exploratory behavior (Li et al., 2023). Mice were placed individually in an open box (60 cm × 60 cm × 40 cm) with a floor divided into 25 equal squares and black-painted walls. Horizontal activity was defined as the number of times a mouse crossed into a new square with all three paws fully entering, whereas vertical activity was defined as the number of times a mouse reared on its hind legs (either free-standing or climbing the wall). Each mouse was observed for 3 min, and both horizontal and vertical activities were recorded. The entire testing process was video-recorded, and the environment was kept quiet to avoid disturbances. After each trial, the box was cleaned with 75% ethanol and any feces were removed to prevent influencing subsequent tests (Luo et al., 2020).

### Lung wet/dry (W/D) weight ratio measurement

Lung tissues were collected 6 h after LPS administration, immediately weighed to obtain the wet weight, and then dried in an oven at 55 °C for 72 h to determine the dry weight. Pulmonary edema was assessed by calculating the wet-to-dry (W/D) weight ratio (Li et al., 2019).

### Hematoxylin and eosin(H&E) staining

Following anesthesia with isoflurane and euthanasia, lung tissues were harvested and fixed in 4% paraformaldehyde. After fixation, the tissues were embedded in paraffin and sectioned for staining. Sections were stained with hematoxylin and eosin (H&E), sealed, and imaged using a Pannoramic Scan digital slide scanner (3DHISTECH, Budapest, Hungary). Lung injury was scored independently by two blinded pathologists based on five histopathological parameters. The severity of injury was graded on a scale from 0 to 4: 0, no lesion; 1, mild inflammation without necrosis; 2, moderate inflammation with occasional necrotic cells; and 4, severe inflammation characterized by frequent necrosis and edema (Lv et al., 2023).

### Immunohistochemistry

Immunohistochemical analysis was performed to assess the expression of F4/80, Ly6G, and MPO in lung tissues. Paraffin-embedded sections underwent heat-induced antigen retrieval and were blocked with 10% goat serum for 30 min at 37 °C. The sections were then incubated overnight at 4 °C with primary antibodies (1:50 dilution). After washing with PBS, HRP-conjugated secondary antibodies were applied and incubated for 3 h at room temperature. Fresh DAB substrate was used for color development, followed by counterstaining with hematoxylin. Finally, the sections were dehydrated, mounted, and imaged under a light microscope.

### Measurement of malondialdehyde (MDA) and glutathione peroxidase (GSH-PX) in lung tissues

To evaluate lipid peroxidation and antioxidant enzyme activities in lung tissues, homogenates were prepared in extraction buffer. MDA and GSH-PX levels were measured using commercial assay kits according to the manufacturer’s instructions.

### Cell culture

Bone marrow-derived macrophages (BMDMs) were isolated from the femurs and tibias of wild-type mice. A small hole was created at the bottom of a 0.6 mL microcentrifuge tube using a 5 mL syringe needle. After removal of the epiphyses, the bones were placed in the prepared tube nested inside a 1.5 mL microcentrifuge tube, and the bone marrow was collected by centrifugation at 10,000 × g for 30 s. Primary BMDMs were cultured at 37 °C in DMEM supplemented with 20% fetal bovine serum (FBS), 25 ng/mL M-CSF, and 1% penicillin-streptomycin (P/S) for 7 days. The culture medium was refreshed on day 3 and replaced on day 5. The mouse macrophage cell line J774A.1 was maintained in DMEM supplemented with 10% FBS and 1% P/S.

### Cytotoxicity assay

The cytotoxicity of CNS toward J774A.1 macrophages was evaluated using the CCK-8 assay. Cells were treated with CNS at concentrations of 2.5, 5, 10, 20, 30, 40, 80, and 100 μM for 12 or 24 h, followed by incubation with CCK-8 reagent (10 μL per well) for 30 min at 37 °C. Absorbance was measured at 450 nm to determine cell viability.

### Inflammasome activation

To stimulate the NLRP3 inflammasome, BMDMs or J774A.1 cells were pretreated with CNS (10, 20, or 40 μM) for 6 h, followed by priming with LPS (100 ng/mL for BMDMs and 500 ng/mL for J774A.1 cells) for 4 h. Cells were then stimulated with nigericin (10 μM for BMDMs and 20 μM for J774A.1 cells) or ATP (4 mM) for 1 h unless otherwise specified (Supplementary Figure S1).

#### Cell death assays

Inflammatory cell death was assessed by staining cells with propidium iodide (PI, 4 μg/mL) and Hoechst 33,342 (5 μg/mL) for 10 min. Images of pyroptotic cells were captured using an INCell Analyzer 2500 equipped with a High-Content Analysis system (GE Healthcare, Pittsburgh, PA, USA).

### ELISA

Conditioned media or serum samples were collected, and mouse IL-1β levels were measured using commercial ELISA kits according to the manufacturer’s instructions (Proteintech, Chicago, IL, USA).

### Immunoblotting

Immunoblotting was performed to detect protein expression in lung tissues, BMDMs, J774A.1 cells, and culture supernatants. As previously described (Pan et al., 2020; Zhu et al., 2019), an equal volume of culture medium was collected and concentrated to detect secreted caspase-1 p10. Protein samples were separated by SDS-PAGE and transferred onto nitrocellulose membranes. After overnight incubation at 4 °C with primary antibodies, membranes were incubated with appropriate horseradish peroxidase (HRP)-conjugated secondary antibodies for 1 h at room temperature. Protein bands were visualized using an SCG-W2000 automatic chemiluminescence imaging system (Servicebio, Shanghai, China) and analyzed with AIWBwell™ software.

### Confocal microscopy

To observe ASC speck formation, BMDMs were fixed with 4% paraformaldehyde for 20 min and permeabilized with 0.5% Triton X-100 for 20 min. Nonspecific binding sites were blocked with 10% goat serum. Cells were then incubated overnight at 4 °C with anti-ASC antibody (1:100), followed by Alexa Fluor 488-conjugated secondary antibody for 1 h and counterstaining with Hoechst 33,342 for 10 min. Confocal images were acquired using an Olympus FV3000 confocal microscope (Tokyo, Japan) and processed with the manufacturer’s software. For detection of Caspase-1 p10, lung tissue sections were incubated with anti-Caspase-1 p10 antibody (1:150) and counterstained with Hoechst. To assess the co-localization of Nrf2 and nuclei, lung sections were incubated with anti-Nrf2 antibody (1:150) followed by Hoechst staining.

### RNA sequencing and bioinformatics analyses

Total RNA was extracted from lung tissues after LPS stimulation using TRIzol reagent. Samples meeting quality control standards were subjected to RNA sequencing (RNA-Seq). Sequencing quality was assessed using FastQC (v0.11.5), and low-quality reads were filtered using NGSQC (v2.3.3). Gene expression was quantified with StringTie (v1.3.3b), and differentially expressed genes were identified for downstream analysis.

### Statistical analysis

All data were analyzed using GraphPad Prism 8.0.2 software. For experiments involving multiple groups with a single independent variable, data were first tested for homogeneity of variance. When variances were equal, one-way ANOVA followed by Bonferroni’s *post hoc* test was applied; when variances were unequal, Welch’s ANOVA followed by Games-Howell *post hoc* test was used. For experiments with multiple groups and two independent variables, two-way ANOVA was performed. Survival analysis was conducted using the log-rank (Mantel-Cox) test. Data are expressed as the mean ± standard deviation (SD), with error bars representing SD.

## Results

### CNS administration attenuates LPS-induced ALI

**FIGURE 1:**
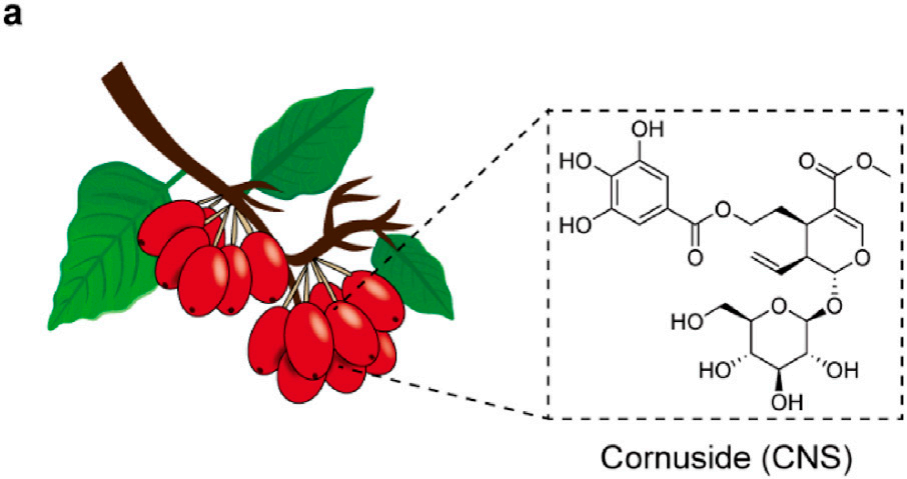
Chemical structure of Cornuside (CNS).

**FIGURE 2:**
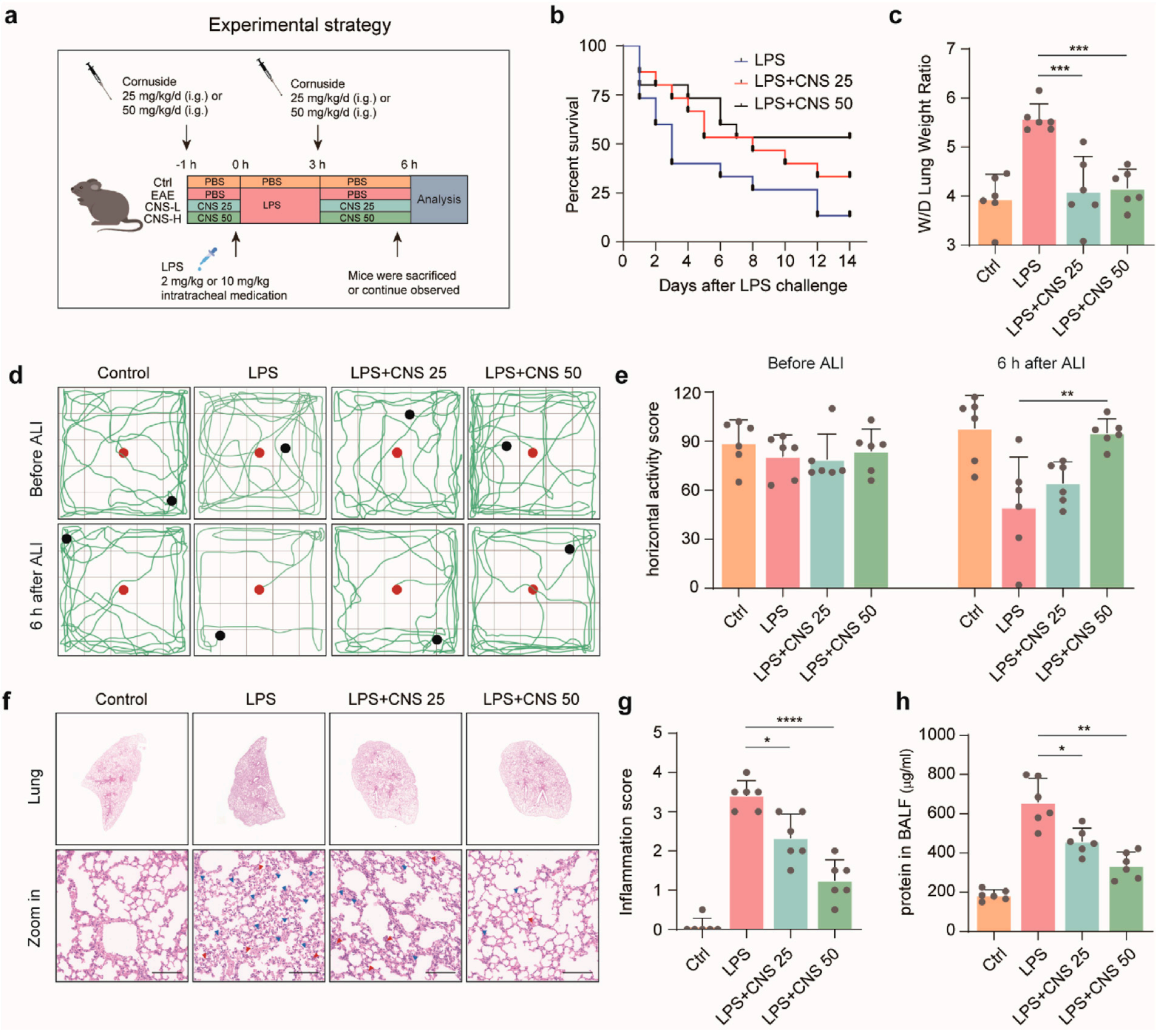
CNS administration attenuates LPS-induced ALI. **(a)** Schematic illustration of the experimental design in mice. **(b)** Survival curves of mice intratracheally injected with LPS (10 mg/kg) with or without CNS treatment (25 or 50 mg/kg; n = 15 per group). **(c)** Lung wet-to-dry weight ratio (n = 6 per group). **(d,e)** Open field test assessing the locomotor activity of mice; red dots indicate starting points and black dots indicate ending points. Quantification of horizontal activity is shown (n = 6 per group). **(f,g)** Representative images of lung tissue stained with H&E and corresponding inflammation scores. Red arrowheads indicate inflammatory cell infiltration or alveolar hemorrhage and blue arrowheads indicate alveolar structural damage (n = 6 per group). Scale bar, 100 μm **(h)** Total protein concentration in bronchoalveolar lavage fluid (BALF) was measured (n = 6 per group). Data are presented as mean ± SD. (*P < 0.05, **P < 0.01, ***P < 0.001, ****P < 0.0001).

To investigate the physiological effects of CNS (Figure 1), a mouse model of LPS-induced ALI was established (Figure 2a). The survival rates of each group are presented in Figure 2b. CNS administration significantly improved the survival of ALI mice compared with the untreated ALI group. Pulmonary edema was evaluated by measuring the lung wet/dry (W/D) weight ratio. As shown in Figure 2c, the W/D ratio was markedly elevated in the ALI group relative to the control group, indicating severe edema following LPS challenge. Pretreatment with CNS (25 and 50 mg/kg) significantly lowered the W/D ratio, suggesting attenuation of pulmonary edema. The open field test was performed to assess spontaneous locomotion and exploratory behavior in ALI mice. Mice in the ALI group displayed reduced horizontal and vertical activity, whereas CNS-treated mice showed a dose-dependent improvement in horizontal movements (Figures 2d,e). Although vertical activity did not differ significantly between the ALI and CNS-treated groups, a trend toward increased rearing was observed (Supplementary Figure S2). Histological analyses further supported the protective effect of CNS. Macroscopic and histopathological evaluations revealed that LPS caused pronounced pathological changes, including inflammatory cell infiltration and disruption of alveolar architecture, whereas CNS treatment markedly ameliorated these lesions (Figures 2f,g). In addition, BALF protein concentration-an indicator of pulmonary vascular permeability-was significantly increased in the ALI group but was substantially reduced by CNS treatment (Figure 2h), indicating improved barrier integrity. Collectively, these findings demonstrate that CNS administration protects against LPS-induced ALI by reducing pulmonary inflammation and edema.

### The potential mechanism of CNS in LPS-induced ALI

**FIGURE 3:**
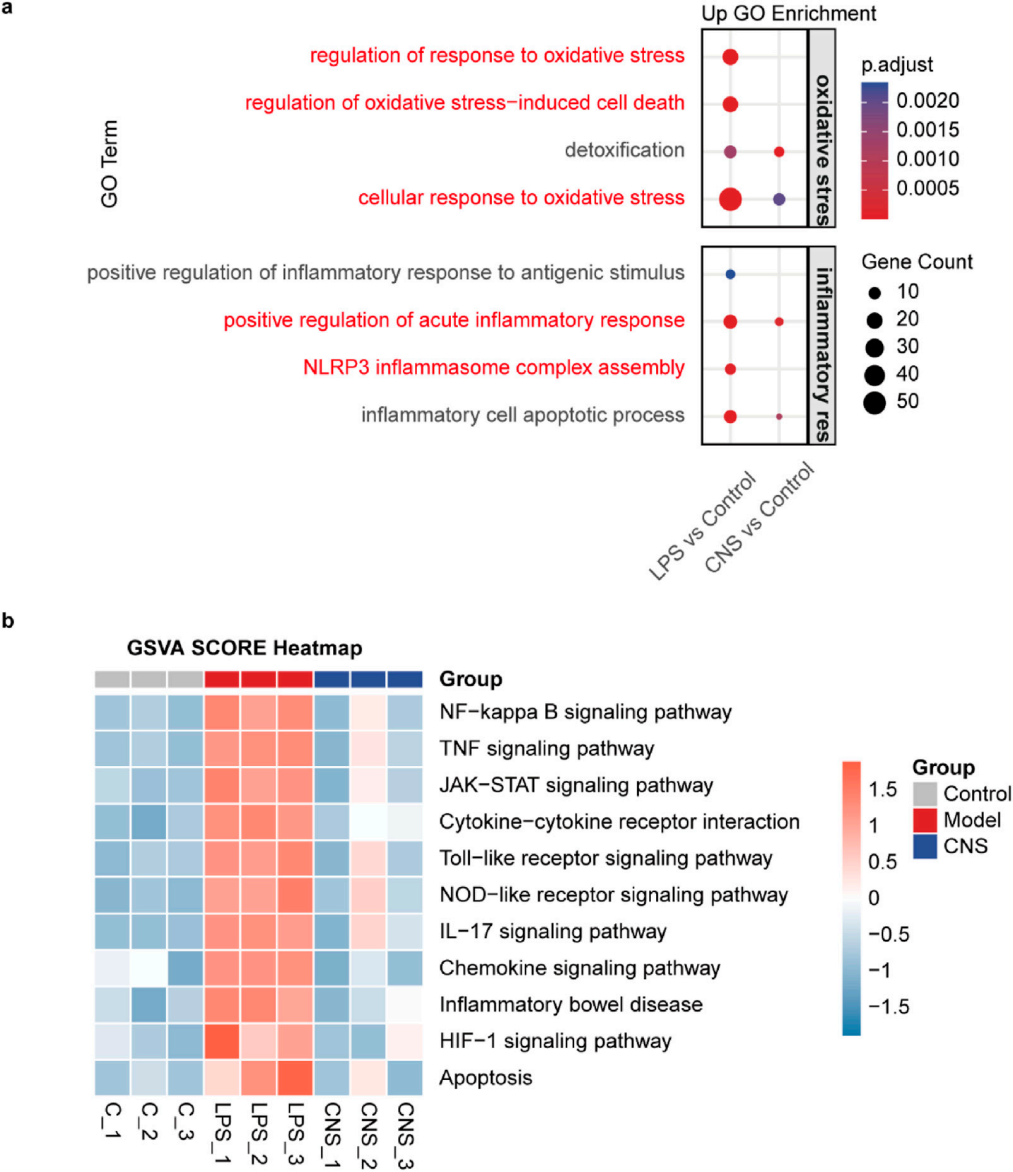
The potential mechanism of CNS in LPS-induced ALI. **(a)** Gene Ontology (GO) enrichment analysis of differentially expressed (DE) mRNAs in lung tissue. Dot color indicates enrichment significance, and dot size represents the number of genes involved (n = 3 per group). **(b)** Gene Set Variation Analysis (GSVA) of different samples (n = 3 per group).

Based on these findings, CNS administration was confirmed to alleviate LPS-induced ALI. To further explore the underlying mechanisms and identify genes significantly regulated by CNS during LPS challenge, RNA-sequencing analysis was performed on lung tissues. Functional enrichment analysis was conducted using the R package clusterProfiler (v4.6.1), with Gene Ontology (GO) enrichment performed via the enrichGO function. The complete list of significantly enriched GO terms, along with gene counts and adjusted P values, is provided in Supplementary Tables S1, S2. As shown in Figure 3a, Gene Ontology (GO) biological process analysis revealed that differentially expressed genes (DEGs) in the model group were significantly enriched in pathways associated with oxidative stress regulation, oxidative stress-induced cell death, cellular response to oxidative stress, positive regulation of the acute inflammatory response, and NLRP3 inflammasome complex assembly compared to the control group. Importantly, CNS treatment largely reversed these alterations. Gene Set Variation Analysis (GSVA) further demonstrated that multiple inflammation-related pathways, including the NF-kappa B signaling pathway, TNF signaling pathway, Cytokine-cytokine receptor interaction, and Toll-like receptor signalling pathway, were strongly activated in the model group. Notably, CNS treatment markedly downregulated these inflammatory pathways relative to the model group, suggesting a key role for CNS in suppressing the inflammatory response (Figure 3b).

### CNS inhibits alveolar inflammatory cell aggregation and expression of inflammatory factors

**FIGURE 4:**
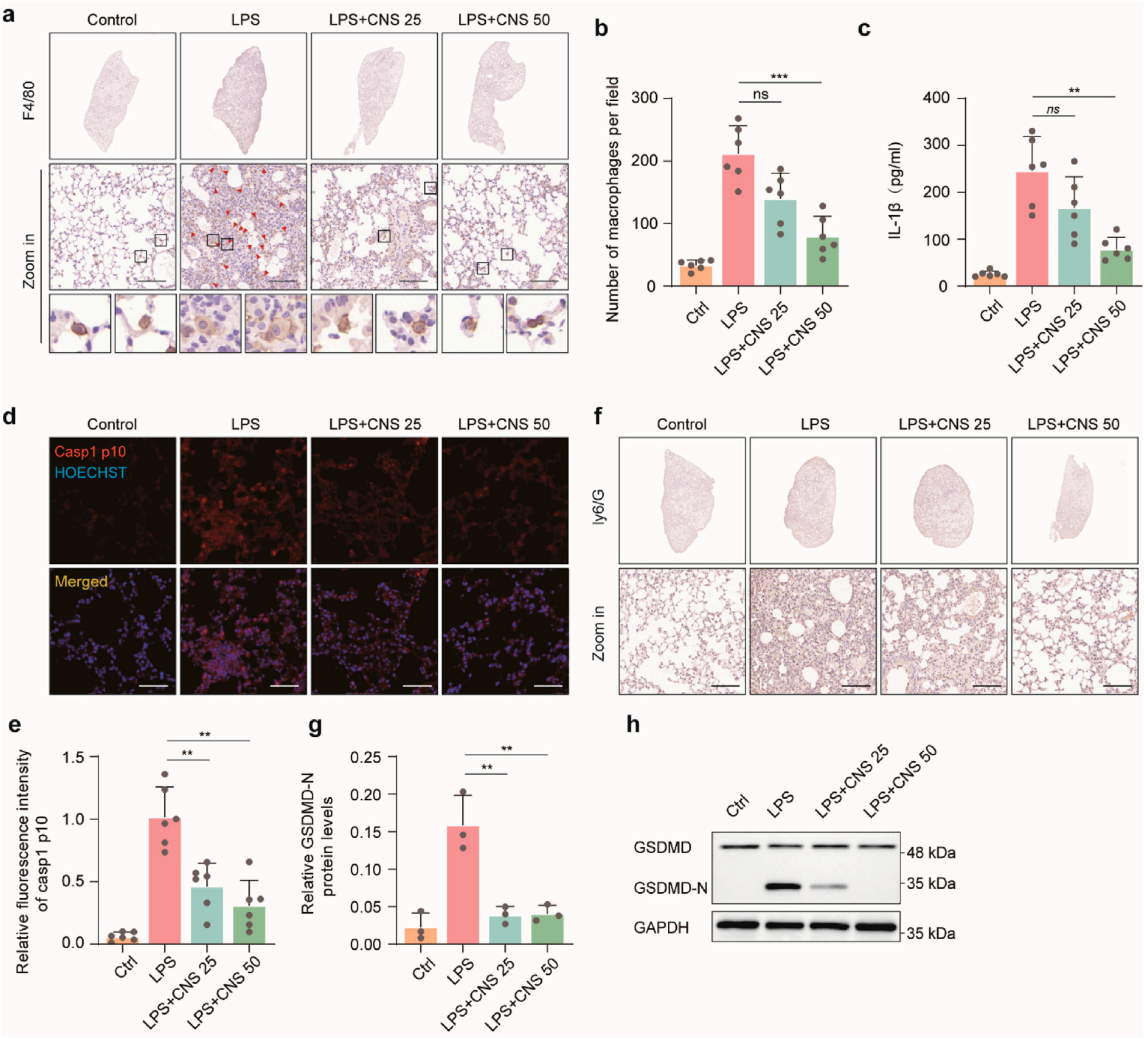
CNS inhibits alveolar inflammatory cell aggregation and expression of inflammatory factors. **(a,b)** Immunohistochemical staining of F4/80 in lung tissues and quantification of alveolar macrophages. Six randomly selected fields per sample were analyzed (n = 6 per group). Scale bar, 100 μm. **(c)** Cytokine (IL-1β) concentrations measured in BALF (n = 6 per group). **(d,e)** Confocal microscopy images of lung tissues stained for caspase-1 p10 and counterstained with Hoechst. Quantification of relative fluorescence intensity of caspase-1 p10 (n = 6 per group). Scale bar, 50 μm. **(f)** Immunohistochemical staining of Ly6G in lung tissues. Scale bar, 25 μm **(g,h)** Western blot analysis of GSDMD expression levels in lung tissues (n = 3 per group). Data are presented as mean ± SD (**P < 0.01, ***P < 0.001; ns, not significant).

Alveolar macrophages-the most abundant innate immune cells in the distal lung parenchyma-play pivotal roles in maintaining homeostasis, defending against pathogens, and orchestrating both the initiation and resolution of pulmonary immune responses (Zhang et al., 2025). F4/80, a well-established macrophage marker, was used to identify alveolar macrophages. Compared with the control group, the number of F4/80-positive macrophages was markedly increased in the model group, and this increase was reversed by CNS treatment. Morphological observation revealed pronounced swelling of alveolar macrophages in the model group, with cell sizes two to three times larger than those in controls, pale cytoplasm and, in some cases, indistinct cell-membrane boundaries; these pathological alterations were alleviated by CNS administration (Figures 4a,b). ELISA and immunohistochemistry showed that IL-1β levels in lung tissue were significantly elevated in the LPS group, whereas CNS treatment reduced IL-1β levels in BALF in a dose-dependent manner (Figure 4c). Immunofluorescence analysis further demonstrated that caspase-1 p10 expression was markedly upregulated in the lungs of the model group, and this increase was effectively suppressed by CNS treatment (Figures 4d,e). Ly6G, a key neutrophils surface marker, was used to evaluate neutrophil infiltration. Immunohistochemical analysis revealed significant neutrophil aggregation in the model group, which was substantially diminished following CNS administration (Figure 4f). In addition, Western blot analysis showed increased expression of the cleaved form of gasdermin D (GSDMD-N) in the model group, whereas CNS treatment significantly reduced GSDMD-N levels (Figures 4g,h).

### CNS inhibits pyroptosis *in vitro*

**FIGURE 5:**
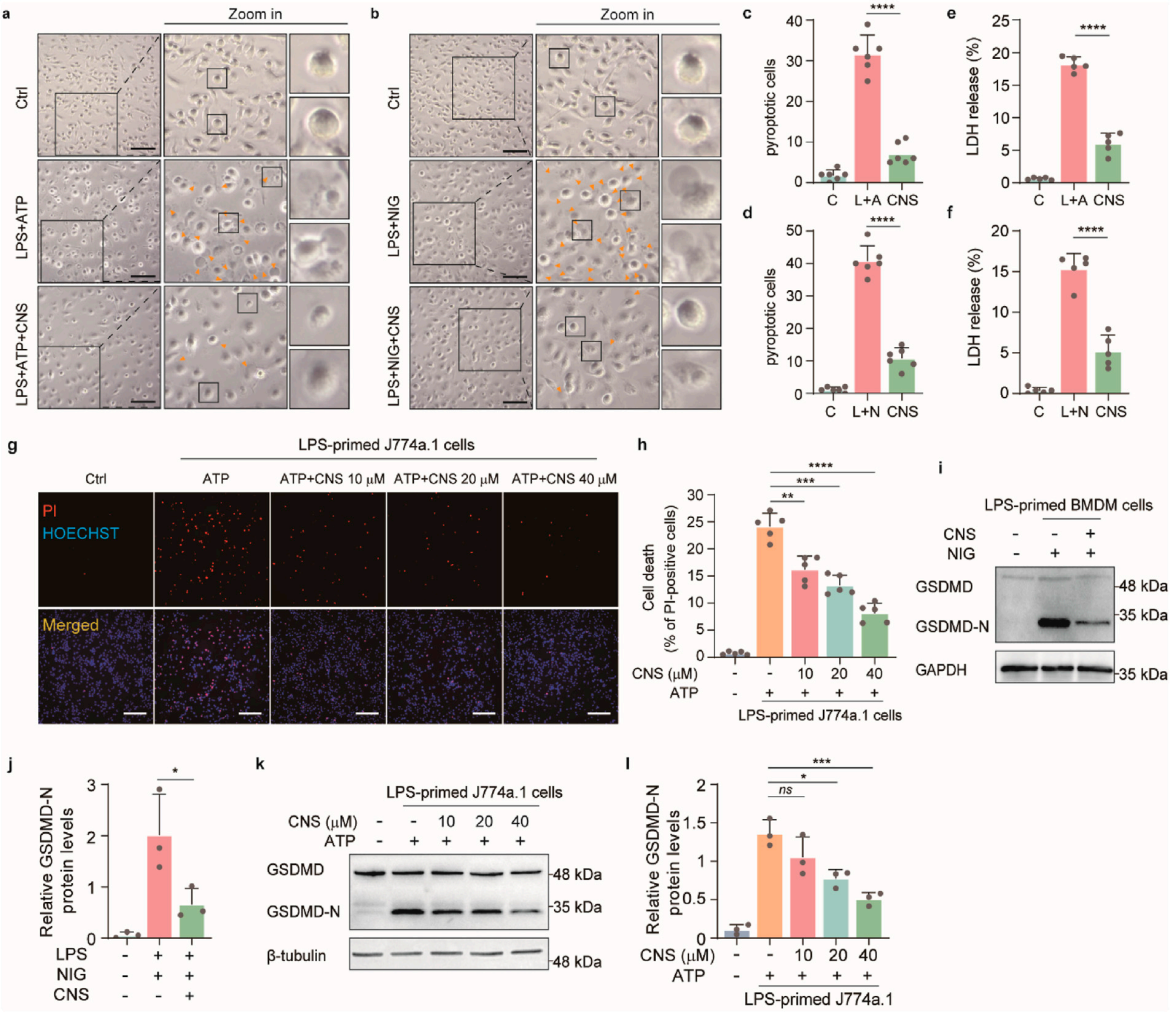
CNS inhibits pyroptosis *in vitro*. **(a-d)** Bright-field images of LPS-primed BMDMs stimulated with ATP or nigericin, with or without CNS pretreatment (arrowheads indicate pyroptotic cells). Quantification of the percentage of pyroptotic cells (%). Scale bar, 100 μm. Six randomly selected fields per sample were analyzed (n = 6 per group). **(e, f)** Effects of CNS treatment on lactate dehydrogenase (LDH) release in BMDMs (n = 5 per group). **(g, h)** Fluorescence images of J774A.1 cells stained with propidium iodide (PI) and Hoechst 33,342. Quantification of PI-positive cells was based on six randomly selected fields (one field per well; n = 5 per group). scale bar, 250 μm. **(i-l)** Western blot analysis of the N-terminal cleavage product of gasdermin D (GSDMD-N) in BMDMs and J774A.1 cells (n = 3 per group). Data are presented as mean ± SD (*P < 0.05, **P < 0.01, ***P < 0.001, ****P < 0.0001; ns, not significant).

Pyroptosis, a form of inflammatory programmed cell death, plays a critical role in the innate immune response and contributes to the progression of numerous inflammatory diseases. This process is characterized by cell swelling, plasma-membrane rupture, and the release of large amounts of inflammatory mediators, thereby amplifying inflammation (Jiang et al., 2023). In the mouse ALI model, CNS markedly improved the abnormal morphology of pulmonary macrophages. We therefore examined the effects of CNS on pyroptosis *in vitro*. To evaluate the potential protective effect of CNS, a CCK-8 assay was first used to assess the cytotoxicity of various CNS concentrations on the mouse macrophage cell line J774A.1 at 12 and 24 h. Based on these results, low-toxicity concentrations (10 μM, 20 μM, and 40 μM) were selected for subsequent experiments (Supplementary Figure S3a,b). Light-microscopy observations revealed that BMDMs displayed typical pyroptotic features after stimulation with LPS-ATP or LPS-nigericin, including cell swelling, membrane blebbing, and, in severe cases, complete membrane rupture and cell disintegration. Notably, CNS treatment markedly alleviated these morphological changes in BMDMs (Figures 5a–d). Consistent with these findings, CNS significantly suppressed LDH release induced by ATP and nigericin stimulation compared with the model group (Figures 5e,f), indicating reduced membrane damage. Propidium iodide (PI) staining was then used to quantify membrane integrity. PI is a fluorescent dye that penetrates cells with compromised membranes and binds to double-stranded DNA, emitting red fluorescence; thus, PI-positive cells indicate membrane disruption and pyroptotic death. As shown in Figures 5g,h, the proportion of PI-positive J774A.1 cells was markedly increased in the LPS + ATP group relative to controls, whereas CNS treatment reduced the percentage of PI-positive cells in a dose-dependent manner. Similar trends were observed in BMDMs stimulated with ATP or nigericin, where CNS likewise decreased the proportion of PI-positive cells (Supplementary Figure S4c,d). Furthermore, Western blot analysis demonstrated that CNS treatment significantly reduced the expression of cleaved gasdermin D (GSDMD-N) in cells stimulated with ATP or nigericin compared with the model group (Figures 5i–l), further supporting the inhibitory effect of CNS on pyroptosis.

### CNS attenuates NLRP3 inflammasome activation *in vitro*

**FIGURE 6:**
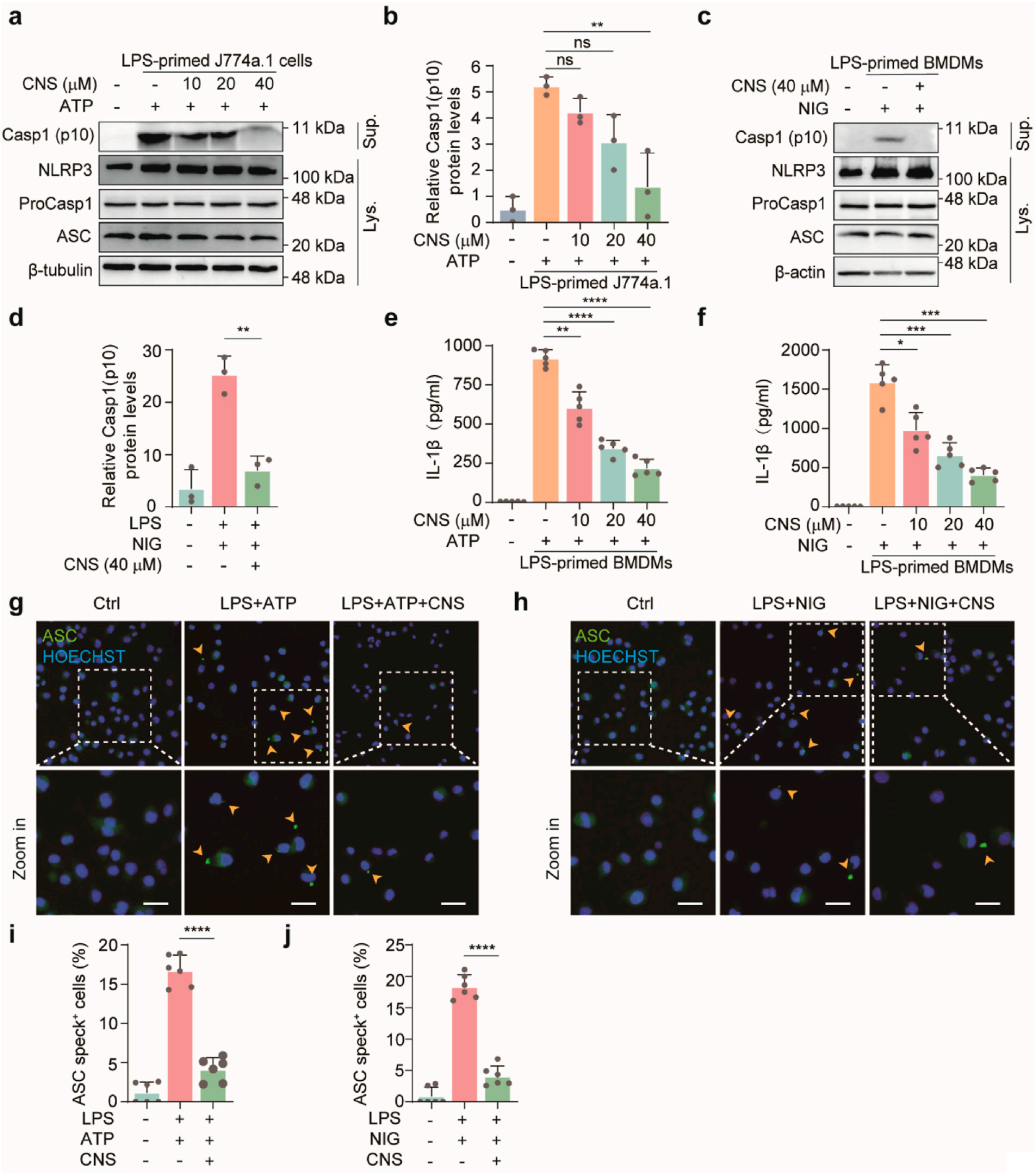
CNS attenuates NLRP3 inflammasome activation *in vitro*. **(a-d)** Western blot analysis of NLRP3, pro-caspase-1, ASC (apoptosis-associated speck-like protein containing a CARD), and cleaved caspase-1 (Casp1-p10) expression in cell lysates and culture supernatants of J774A.1 cells and BMDMs (n = 3 per group). **(e–f)** Effect of CNS treatment on interleukin-1β (IL-1β) production in the culture supernatants of BMDMs (n = 5 per group). **(g–j)** Immunofluorescence images showing the subcellular distribution of ASC (green) in BMDMs. Nuclei were counterstained with Hoechst 33,342 (blue); arrowheads indicate ASC specks. Scale bar, 20 μm. Quantification of ASC specks was performed from six random fields (one field per well) (n = 6 per group). Data are presented as mean ± SD (*P < 0.05, **P < 0.01, ***P < 0.001, ****P < 0.0001; ns, not significant).

Upon activation, the NLRP3 inflammasome promotes the cleavage of pro-IL-1β and GSDMD by activated caspase-1 (caspase-1 p10), generating mature IL-1β and the active GSDMD-N fragment. GSDMD-N subsequently forms pores in the plasma membrane, facilitating the release of inflammatory mediators and triggering pyroptotic cell death. Because our previous morphological and molecular analyses showed that CNS inhibited GSDMD-N expression and improved cellular morphology, we next examined whether CNS also modulates upstream NLRP3 inflammasome activation *in vitro*. Western blot analysis was performed to detect the expression levels of cleaved caspase-1 (caspase-1 p10) in the culture supernatants of J774A.1 cells and BMDMs, as well as the levels of pro-caspase-1, NLRP3, and ASC in cell lysates, to assess the regulatory effect of CNS on inflammasome activation. In J774A.1 cells, stimulation with LPS-ATP markedly induced NLRP3 expression and also promoted the release of caspase-1 p10 into the culture supernatant (Figures 6a,b; Supplementary Figure S4a-c). Notably, CNS treatment significantly reduced the release of cleaved caspase-1 in a concentration-dependent manner. Similarly, in BMDMs, LPS-nigericin stimulation robustly activated the inflammasome, as evidenced by elevated NLRP3 levels in the lysates and increased cleaved caspase-1 in the supernatant, whereas CNS treatment effectively suppressed caspase-1 cleavage and release under stimulation conditions (Figures 6c,d; Supplementary Figure S4d-f). Consistent with these results, CNS treatment also reduced IL-1β secretion induced by NLRP3 activators, as measured by ELISA (Figures 6e,f). ASC, a key adaptor for inflammasome assembly, oligomerizes upon NLRP3 activation to form perinuclear aggregates (“ASC specks”) that recruit and activate downstream signaling components. Concordant with its inhibitory effect on inflammasome activation, CNS treatment markedly reduced ASC speck formation in LPS-primed BMDMs stimulated with ATP or nigericin (Figures 6g–j). Collectively, these findings suggest that CNS effectively attenuates NLRP3 inflammasome activation.

### CNS regulates the Keap1-Nrf2 pathway and reduces oxidative stress in the lungs

**FIGURE 7:**
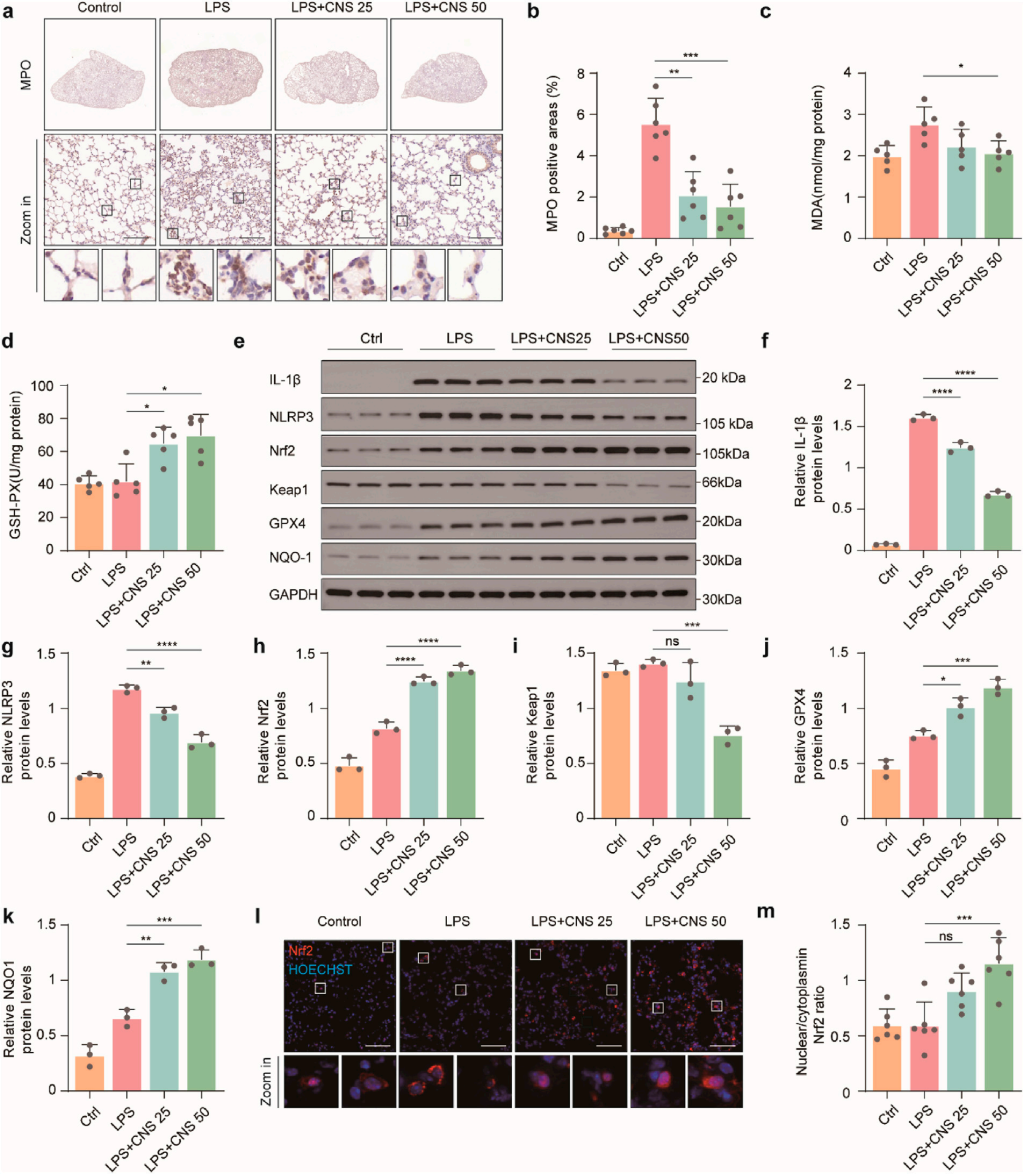
CNS regulates the Keap1-Nrf2 pathway and reduces oxidative stress in the lungs. **(a-b)** Immunohistochemical staining of myeloperoxidase (MPO) in lung tissues (n = 6 per group). **(c)** Malondialdehyde (MDA) levels in lung tissues measured with an MDA assay kit (n = 5 per group). **(d)** Glutathione peroxidase (GSH-PX) levels using a GSH assay kit (n = 5 per group). **(e-k)** Western blot analysis of IL-1β, NLRP3, Keap1, Nrf2, GPX4, and NQO1 expression in lung tissues (n = 3 per group). **(l)** Immunofluorescence analysis showing Nrf2 expression and nuclear translocation. scale bar, 50 μm. Data are presented as mean ± SD (*P < 0.05, **P < 0.01, ***P < 0.001, ****P < 0.0001; ns, not significant).

Although numerous stimuli are known to activate the NLRP3 inflammasome, direct ligand binding to NLRP3 has not been demonstrated (Chen and Chen, 2018). Instead, activation is generally thought to occur through common upstream signaling events within the cell. Reactive oxygen species (ROS), generated by enzymes such as myeloperoxidase (MPO), are key contributors to NLRP3 inflammasome activation. To determine whether CNS modulates oxidative stress in LPS-induced ALI, MPO expression was assessed by immunohistochemistry. As shown in Figures 7a,b, MPO expression was significantly elevated in the lungs of the model group, whereas CNS treatment dose-dependently reduced MPO levels. Malondialdehyde (MDA), a terminal product of lipid peroxidation formed when ROS attack polyunsaturated fatty acids, serves as a reliable indicator of oxidative stress. As depicted in Figure 7c, MDA content in lung tissue was markedly increased in the model group, whereas CNS treatment significantly attenuated this increase. In parallel, GSH-PX, a key antioxidant enzyme that eliminates ROS and maintains redox homeostasis, was significantly increased by CNS treatment (Figure 7d), indicating enhanced antioxidant capacity. Given the central role of the Keap1-Nrf2 pathway in regulating cellular antioxidant responses, we next evaluated the effect of CNS on this pathway. Under oxidative stress, activation of the Keap1-Nrf2 axis allows Nrf2 to translocate into the nucleus, where it drives transcription of downstream antioxidant genes such as glutathione peroxidase 4 (GPX4) and NAD(P)H quinone dehydrogenase 1 (NQO1), thereby mitigating ROS-mediated activation of the NLRP3 inflammasome. Western blot analysis confirmed that CNS treatment increased Nrf2 and its downstream antioxidant proteins GPX4 and NQO1 in lung tissue, accompanied by a reduction in Keap1 expression (Figures 7e–k). Immunofluorescence further demonstrated that CNS enhanced both Nrf2 expression its nuclear translocation (Figures 7l,m). Collectively, these findings indicate that CNS reduces oxidative stress in LPS-induced lung injury by strengthening antioxidant defenses through activation of the Keap1-Nrf2 signaling pathway.

## Discussion

ALI and its severe form, acute respiratory distress syndrome (ARDS), are life-threatening respiratory disorders characterized by excessive inflammation, epithelial barrier disruption, pulmonary edema, and respiratory dysfunction, posing a major clinical challenge (Cai et al., 2025; Gu et al., 2024). To date, no specific or highly effective pharmacological therapy has been identified, and current treatments remain largely supportive, underscoring the urgent need for novel therapeutic strategies (Verma et al., 2025). In the present study, we demonstrate for the first time that CNS, an iridoid glycoside from *C*. *officinalis*, improves survival and alleviates pulmonary edema, inflammation, and locomotor deficits in LPS-induced ALI, establishing CNS as a promising natural compound for inflammatory lung injury.

Alveolar macrophages, which reside within the alveolar airspaces and are closely associated with the lung epithelium, play a crucial role in maintaining pulmonary homeostasis by clearing cellular debris and and resolving inflammation (Kumar, 2020). Under pathological conditions, however, dysregulated activation of these cells drives the development and progression of ALI (Ne et al., 2019). Instead of promoting resolution, overactivated alveolar macrophages produce excessive pro-inflammatory cytokines, thereby amplifying lung inflammation, disrupting the alveolar-capillary barrier, and exacerbating tissue injury (Jin et al., 2020; Hu et al., 2021; Wu et al., 2021). In our study, F4/80 staining revealed that alveolar macrophages in the model group were markedly swollen, exhibiting enlarged cell size, pale cytoplasm, and indistinct membrane boundaries-morphological changes that were effectively reversed by CNS treatment. Combined with our RNA-seq findings, these observations suggested that the alveolar macrophages might have undergone pyroptosis. During pyroptosis, activated caspase-1 cleaves gasdermin D (GSDMD), a 242-amino-acid protein with an N-terminal pore-forming domain (GSDMD-N) and a C-terminal inhibitory domain (GSDMD-C). The released GSDMD-N translocates to the plasma membrane, forms pores, and disrupts membrane integrity, leading to cell swelling and the release of pro-inflammatory cytokines (Ding et al., 2016; Qiu et al., 2017). Consistent with this mechanism, our biochemical data show that CNS markedly reduces IL-1β release, caspase-1 p10 formation, GSDMD cleavage, and ASC speck assembly both *in vivo* and *in vitro*, indicating effective inhibition of NLRP3 inflammasome activation and pyroptotic cell death. These findings are consistent with previous work identifying the NLRP3 inflammasome as a central driver of ALI pathogenesis.

Our transcriptomic and biochemical data also indicate that CNS alleviates oxidative stress while suppressing NLRP3 inflammasome activation. These findings fit well with a broader network of redox-sensitive inflammatory signaling that has been described in LPS-induced models. Previous studies have shown that pathways such as TLR4, NF-κB, p38 MAPK, and P2X7 receptor signaling lie upstream of both inflammasome activation and intrinsic apoptosis, while anti-oxidative defenses (Nrf2, HO-1, Bcl-2) counteract these signals (Ulaş et al., 2025; Shen et al., 2024; Ozkanlar et al., 2025; Huang et al., 2020). In particular, TLR4 activation by LPS triggers NF-κB signaling, driving the transcription of pro-inflammatory cytokines such as TNF-α, IL-1β, and IL-6. This inflammatory cascade not only promotes ROS production through mitochondrial and NADPH oxidase pathways but also provides upstream signals for NLRP3 inflammasome assembly (Ozkanlar et al., 2025; Shen et al., 2021; Zhou et al., 2023). Excessive ROS, in turn, feeds back to further activate NF-κB and facilitates P2X7 receptor-mediated potassium efflux, both of which potentiate NLRP3 activation and pyroptotic cell death. Conversely, the Keap1-Nrf2 antioxidant axis acts as a critical counter-regulatory pathway. Under oxidative stress, Nrf2 escapes Keap1 repression and translocates to the nucleus, inducing HO-1 and other antioxidant enzymes (such as GPX4 and NQO1) (Zhang J. et al., 2022; Baird and Yamamoto, 2020). Activation of this pathway reduces intracellular ROS, thereby indirectly suppressing NF-κB activity, limiting NLRP3 activation, and dampening downstream cytokine release. Importantly, the reduction in ROS also impacts apoptotic signaling. Mitochondrial ROS accumulation can lower anti-apoptotic Bcl-2 levels, enhance Bax translocation, and activate caspase-3/9, driving intrinsic apoptosis (Ozkanlar et al., 2025; Yang R et al., 2022). In our study, CNS treatment dose-dependently reduced MPO and MDA levels while increasing GSH-PX activity in lung tissue, indicating attenuation of oxidative stress. Moreover, CNS activated the Keap1-Nrf2 pathway by promoting Nrf2 expression and nuclear translocation, thereby upregulating downstream antioxidant proteins such as NQO1. Although apoptosis was not directly assessed in this work, our transcriptomic data suggest that CNS may also attenuate pro-apoptotic signaling. Together with the observed suppression of pyroptosis, these findings raise the possibility that CNS mitigates ALI by dampening multiple forms of programmed cell death through its antioxidant and anti-inflammasome effects.

Pharmacological activation of Nrf2 has emerged as a therapeutic approach for sepsis-induced ALI. CNS shares this antioxidant mechanism but offers the additional advantage of suppressing NLRP3 inflammasome activation, suggesting a dual mechanism that may provide broader protection. Importantly, CNS is a naturally occurring compound with a favorable safety profile in traditional use, which may facilitate future translational development. In addition, several limitations should be acknowledged. First, only male C57BL/6J mice were used; sex-dependent pharmacological responses remain to be evaluated. Second, apoptosis and other forms of cell death such as ferroptosis were not directly measured, and mechanistic links inferred from transcriptomics require experimental validation. Third, pharmacokinetic and dose–response studies are needed to guide clinical translation. Despite these limitations, our findings provide a mechanistic framework and preclinical evidence for CNS as a potential therapy for ALI and possibly other oxidative stress-related lung diseases, including severe COVID-19.

In conclusion, we provide compelling evidence that CNS protects against LPS-induced ALI by attenuating oxidative stress and suppressing NLRP3 inflammasome activation via activation of the Keap1-Nrf2 pathway. Through these anti-inflammatory and anti-oxidative actions, CNS may also indirectly limit apoptosis. These results elucidate the molecular basis underpinning the anti-inflammatory effects of CNS and lay a scientific foundation for its future development in ALI-and potentially in other oxidative stress-and inflammasome-related diseases.

## Data Availability

The original contributions presented in the study are publicly available. This data can be found here: Genome Sequence Archive (GSA), accession number CRA030589, https://ngdc.cncb.ac.cn/gsa/search?searchTerm=CRA030589.

## References

[B1] Aliberti S. Dela Cruz C. S. Amati F. Sotgiu G. Restrepo M. I. (2021). Community-acquired pneumonia. Lancet 398 (10303), 906–919. 10.1016/S0140-6736(21)00630-9 34481570

[B2] Baird L. Yamamoto M. (2020). The molecular mechanisms regulating the KEAP1-NRF2 pathway. Mol. Cell Biol. 40 (13), e00099-20. 10.1128/MCB.00099-20 32284348 PMC7296212

[B3] Cai Y. Shang L. Zhou F. Zhang M. Li J. Wang S. (2025). Macrophage pyroptosis and its crucial role in ALI/ARDS. Front. Immunol. 16, 1530849. 10.3389/fimmu.2025.1530849 40028334 PMC11867949

[B4] Chen J. Chen Z. J. (2018). PtdIns4P on dispersed trans-Golgi network mediates NLRP3 inflammasome activation. Nature 564 (7734), 71–76. 10.1038/s41586-018-0761-3 30487600 PMC9402428

[B5] Cui X. Yu Y. Yu J. Xu K. Sun X. (2024). Iridoid glycoside cornuside alleviates the symptom of gestational diabetes mellitus by suppressing inflammation and regulating beta cell function. Gynecol. Obstet. Invest 89 (1), 59–68. 10.1159/000534623 37980893

[B6] Dai H. Pan L. Lin F. Ge W. Li W. He S. (2015). Mechanical ventilation modulates toll-like receptors 2, 4, and 9 on alveolar macrophages in a ventilator-induced lung injury model. J. Thorac. Dis. 7 (4), 616–624. 10.3978/j.issn.2072-1439.2015.02.10 25973227 PMC4419314

[B7] Ding J. Wang K. Liu W. She Y. Sun Q. Shi J. (2016). Pore-forming activity and structural autoinhibition of the gasdermin family. Nature 535 (7610), 111–116. 10.1038/nature18590 27281216

[B8] Dolinay T. Kim Y. S. Howrylak J. Hunninghake G. M. An C. H. Fredenburgh L. (2012). Inflammasome-regulated cytokines are critical mediators of acute lung injury. Am. J. Respir. Crit. Care Med. 185 (11), 1225–1234. 10.1164/rccm.201201-0003OC 22461369 PMC3373064

[B9] Fan E. Brodie D. Slutsky A. S. (2018). Acute respiratory distress syndrome: advances in diagnosis and treatment. JAMA 319 (7), 698–710. 10.1001/jama.2017.21907 29466596

[B10] Gao F. Xia S.-L. Wang X.-H. Zhou X.-X. Wang J. (2021). Cornuside I promoted osteogenic differentiation of bone mesenchymal stem cells through PI3K/Akt signaling pathway. J. Orthop. Surg. Res. 16 (1), 397. 10.1186/s13018-021-02508-0 34154621 PMC8218506

[B11] Gu W. Zeng Q. Wang X. Jasem H. Ma L. (2024). Acute lung injury and the NLRP3 inflammasome. J. Inflamm. Res. 17, 3801–3813. 10.2147/JIR.S464838 38887753 PMC11182363

[B12] Hu Q. Lyon C. J. Fletcher J. K. Tang W. Wan M. Hu T. Y. (2021). Extracellular vesicle activities regulating macrophage- and tissue-mediated injury and repair responses. Acta Pharm. Sin. B 11 (6), 1493–1512. 10.1016/j.apsb.2020.12.014 34221864 PMC8245807

[B13] Huang C.-Y. Deng J.-S. Huang W.-C. Jiang W.-P. Huang G.-J. (2020). Attenuation of lipopolysaccharide-induced acute lung injury by hispolon in mice, through regulating the TLR4/PI3K/Akt/mTOR and Keap1/Nrf2/HO-1 pathways, and suppressing oxidative stress-mediated ER stress-induced apoptosis and autophagy. Nutrients 12 (6), 1742. 10.3390/nu12061742 32532087 PMC7352175

[B14] Jiang J. Huang K. Xu S. Garcia J. G. N. Wang C. Cai H. (2020). Targeting NOX4 alleviates sepsis-induced acute lung injury *via* attenuation of redox-sensitive activation of CaMKII/ERK1/2/MLCK and endothelial cell barrier dysfunction. Redox Biol. 36, 101638. 10.1016/j.redox.2020.101638 32863203 PMC7381685

[B15] Jiang Y. Gao S. Chen Z. Zhao X. Gu J. Wu H. (2023). Pyroptosis in septic lung injury: interactions with other types of cell death. Biomed. Pharmacother. 169, 115914. 10.1016/j.biopha.2023.115914 38000360

[B16] Jin S.-H. Sun J.-J. Liu G. Shen L.-J. Weng Y. Li J.-Y. (2020)2023). Nrf2/PHB2 alleviates mitochondrial damage and protects against staphylococcus aureus-induced acute lung injury. MedComm 4 (6), e448. 10.1002/mco2.448 38077250 PMC10701464

[B17] Kuipers M. T. Aslami H. Janczy J. R. van der Sluijs K. F. Vlaar A. P. J. Wolthuis E. K. (2012). Ventilator-induced lung injury is mediated by the NLRP3 inflammasome. Anesthesiology 116 (5), 1104–1115. 10.1097/ALN.0b013e3182518bc0 22531249

[B18] Kumar V. (2020). Pulmonary innate immune response determines the outcome of inflammation during pneumonia and sepsis-associated acute lung injury. Front. Immunol. 11, 1722. 10.3389/fimmu.2020.01722 32849610 PMC7417316

[B19] Li X. Jamal M. Guo P. Jin Z. Zheng F. Song X. (2019). Irisin alleviates pulmonary epithelial barrier dysfunction in sepsis-induced acute lung injury *via* activation of AMPK/SIRT1 pathways. Biomed. Pharmacother. 118, 109363. 10.1016/j.biopha.2019.109363 31545277

[B20] Li Y.-L. Qin S.-Y. Li Q. Song S.-J. Xiao W. Yao G.-D. (2023). Jinzhen oral liquid alleviates lipopolysaccharide-induced acute lung injury through modulating TLR4/MyD88/NF-κB pathway. Phytomedicine 114, 154744. 10.1016/j.phymed.2023.154744 36934667

[B21] Lian W. Wang Z. Zhou F. Yuan X. Xia C. Wang W. (2024). Cornuside ameliorates cognitive impairments *via* RAGE/TXNIP/NF-κB signaling in Aβ1-42 induced alzheimer's disease mice. J. Neuroimmune Pharmacol. 19 (1), 24. 10.1007/s11481-024-10120-2 38780885

[B22] Liu Y. Wang C. Hui T. Yuan Y. Chen S. Li Y. (2025). Cornus officinalis loganin attenuates acute lung injury in mice *via* regulating the PI3K/AKT/NLRP3 axis. J. Ethnopharmacol. 351, 120104. 10.1016/j.jep.2025.120104 40490233

[B23] Long M. E. Mallampalli R. K. Horowitz J. C. (2022). Pathogenesis of pneumonia and acute lung injury. Clin. Sci. (Lond). 136 (10), 747–769. 10.1042/CS20210879 35621124 PMC9429452

[B24] Luo T. Tian H. Song H. Zhao J. Liya A. Fang Y. (2020). Possible involvement of tissue plasminogen Activator/brain-derived neurotrophic factor pathway in anti-depressant effects of electroacupuncture in chronic unpredictable mild stress-induced depression in rats. Front. Psychiatry 11, 63. 10.3389/fpsyt.2020.00063 32153441 PMC7044269

[B25] Lv Y.-W. Du Y. Ma S.-S. Shi Y.-C. Xu H.-C. Deng L. (2023). Proanthocyanidins attenuates ferroptosis against influenza-induced acute lung injury in mice by reducing IFN-γ. Life Sci. 314, 121279. 10.1016/j.lfs.2022.121279 36526043

[B26] Meng Z. Gao M. Wang C. Guan S. Zhang D. Lu J. (2023). Apigenin alleviated high-fat-diet-induced hepatic pyroptosis by Mitophagy-ROS-CTSB-NLRP3 pathway in mice and AML12 cells. J. Agric. Food Chem. 71 (18), 7032–7045. 10.1021/acs.jafc.2c07581 37141464

[B27] Nepal S. Tiruppathi C. Tsukasaki Y. Farahany J. Mittal M. Rehman J. (2019). STAT6 induces expression of Gas6 in macrophages to clear apoptotic neutrophils and resolve inflammation. Proc. Natl. Acad. Sci. U. S. A. 116 (33), 16513–16518. 10.1073/pnas.1821601116 31363052 PMC6697797

[B28] Nguyen N. Xu S. Lam T. Y. W. Liao W. Wong W. S. F. Ge R. (2022). ISM1 suppresses LPS-Induced acute lung injury and post-injury lung fibrosis in mice. Mol. Med. 28 (1), 72. 10.1186/s10020-022-00500-w 35752760 PMC9233842

[B29] Ozkanlar S. Ozkanlar Y. Kara A. Dalkilinc E. (2025). Astaxanthin alleviates lung injury by regulating oxidative stress, inflammatory response, P2X7 receptor, NF-κB, Bcl-2, and Caspase-3 in LPS-induced endotoxemia. Environ. Toxicol. 40 (6), 924–934. 10.1002/tox.24481 39873358 PMC12069755

[B30] Pan H. Lin Y. Dou J. Fu Z. Yao Y. Ye S. (2020). Wedelolactone facilitates Ser/Thr phosphorylation of NLRP3 dependent on PKA signalling to block inflammasome activation and pyroptosis. Cell Prolif. 53 (9), e12868. 10.1111/cpr.12868 32656909 PMC7507381

[B31] Pham T. Rubenfeld G. D. (2017). Fifty years of research in ARDS. The epidemiology of acute respiratory distress syndrome. A 50th birthday review. Am. J. Respir. Crit. Care Med. 195 (7), 860–870. 10.1164/rccm.201609-1773CP 28157386

[B32] Qin Y.-Y. Yu H. Huang Y. Yang X. Li S. Shen A. (2024). Naked gene delivery induces autophagy for effective treatment of acute lung injury in a mouse model. Int. J. Nanomedicine 19, 10801–10818. 10.2147/IJN.S477947 39469449 PMC11514649

[B33] Qiu S. Liu J. Xing F. (2017). Hints' in the killer protein gasdermin D: unveiling the secrets of gasdermins driving cell death. Cell Death Differ. 24 (4), 588–596. 10.1038/cdd.2017.24 28362726 PMC5384029

[B34] Ren W. Sun Y. Zhao L. Shi X. (2024). NLRP3 inflammasome and its role in autoimmune diseases: a promising therapeutic target. Biomed. Pharmacother. 175, 116679. 10.1016/j.biopha.2024.116679 38701567

[B35] Shan M. Wan H. Ran L. Ye J. Xie W. Lu J. (2024). Dynasore alleviates LPS-Induced acute lung injury by inhibiting NLRP3 inflammasome-mediated pyroptosis. Drug Des. Devel Ther. 18, 1369–1384. 10.2147/DDDT.S444408 38681210 PMC11055558

[B36] Shen S. He F. Cheng C. Xu B. Sheng J. (2021). Uric acid aggravates myocardial ischemia-reperfusion injury *via* ROS/NLRP3 pyroptosis pathway. Biomed. Pharmacother. 133, 110990. 10.1016/j.biopha.2020.110990 33232925

[B37] Shen X. He L. Cai W. (2024). Role of lipopolysaccharides in the inflammation and pyroptosis of alveolar epithelial cells in acute lung injury and acute respiratory distress syndrome. J. Inflamm. Res. 17, 5855–5869. 10.2147/JIR.S479051 39228678 PMC11370780

[B38] Shi J.-Z. Zheng X.-M. Zhou Y.-F. Yun L.-Y. Luo D.-M. Hao J.-J. (2022). Cornuside is a potential agent against Alzheimer's disease *via* orchestration of reactive astrocytes. Nutrients 14 (15), 3179. 10.3390/nu14153179 35956355 PMC9370780

[B39] Spychaj R. Kucharska A. Z. Szumny A. Przybylska D. Pejcz E. Piórecki N. (2021). Potential valorization of Cornelian cherry (cornus mas L.) stones: roasting and extraction of bioactive and volatile compounds. Food Chem. 358, 129802. 10.1016/j.foodchem.2021.129802 33933979

[B40] Tao W. Hu Y. Chen Z. Dai Y. Hu Y. Qi M. (2021). Magnolol attenuates depressive-like behaviors by polarizing microglia towards the M2 phenotype through the regulation of Nrf2/HO-1/NLRP3 signaling pathway. Phytomedicine 91, 153692. 10.1016/j.phymed.2021.153692 34411834

[B41] Ulaş N. Üstündağ H. Özkanlar S. Erbaş E. Kara A. Özkanlar Y. (2025). D-carvone attenuates LPS-Induced acute lung injury *via* TLR4/NF-κB and Nrf2/HO-1 signaling pathways in rats. Naunyn Schmiedeb. Arch. Pharmacol. 3 (1), 12215–12225. 10.1007/s00210-025-04024-y 40116872 PMC12449407

[B42] Verma N. Hochhegger B. Mukhopadhyay S. Teixeira E. Silva Torres P. P. Mohammed T.-L. (2025). Acute lung injury. J. Thorac. Imaging. 40 (3) e0820. 10.1097/RTI.0000000000000820 39654323

[B43] Wang L. Chen H. Jiang Y. Liu Z. Wang Q. Zheng X. (2018). Simultaneous determination of 11 high-polarity components from fructus Corni: a quantitative LC-MS/MS method for improved quality control. J. Chromatogr. Sci. 56 (1), 56–64. 10.1093/chromsci/bmx083 29036589

[B44] Wang X. Wu F.-P. Huang Y.-R. Li H.-D. Cao X.-Y. You Y. (2023a). Matrine suppresses NLRP3 inflammasome activation *via* regulating PTPN2/JNK/SREBP2 pathway in sepsis. Phytomedicine 109, 154574. 10.1016/j.phymed.2022.154574 36610161

[B45] Wang L. Yan F. Zhang J. Xiao Y. Wang C. Zhu Y. (2023b). Cornuside improves murine autoimmune hepatitis through inhibition of inflammatory responses. Phytomedicine 120, 155077. 10.1016/j.phymed.2023.155077 37716032

[B46] Wang F. Ge R. Cai Y. Zhao M. Fang Z. Li J. (2025). Oxidative stress in ARDS: mechanisms and therapeutic potential. Front. Pharmacol. 16, 1603287. 10.3389/fphar.2025.1603287 40642004 PMC12241040

[B47] Wei J. Liu Z. Sun H. Xu L. (2024). Perillaldehyde ameliorates lipopolysaccharide-induced acute lung injury *via* suppressing the cGAS/STING signaling pathway. Int. Immunopharmacol. 130, 111641. 10.1016/j.intimp.2024.111641 38368770

[B48] Wu G. Zhu Q. Zeng J. Gu X. Miao Y. Xu W. (2019). Extracellular mitochondrial DNA promote NLRP3 inflammasome activation and induce acute lung injury through TLR9 and NF-κB. J. Thorac. Dis. 11 (11), 4816–4828. 10.21037/jtd.2019.10.26 31903272 PMC6940233

[B49] Wu D. Zhang H. Wu Q. Li F. Wang Y. Liu S. (2021). Sestrin 2 protects against LPS-induced acute lung injury by inducing mitophagy in alveolar macrophages. Life Sci. 267, 118941. 10.1016/j.lfs.2020.118941 33359748

[B50] Xia L. Zhang C. Lv N. Liang Z. Ma T. Cheng H. (2022). AdMSC-derived exosomes alleviate acute lung injury *via* transferring mitochondrial component to improve homeostasis of alveolar macrophages. Theranostics 12 (6), 2928–2947. 10.7150/thno.69533 35401830 PMC8965475

[B51] Xiang F. Li X. Hu W. (2024). Cornuside ameliorates diabetic nephropathy possibly by regulating angiogenesis and MAPK signaling. Tohoku J. Exp. Med. 1, 87–95. 10.1620/tjem.2024.J112 39443135

[B52] Yang J. Yang J. Huang X. Xiu H. Bai S. Li J. (2022). Glibenclamide alleviates LPS-Induced acute lung injury through NLRP3 inflammasome signaling pathway. Mediat. Inflamm. 2022:8457010. 10.1155/2022/8457010 35185385 PMC8856806

[B53] Yang R R. Yang H. Li W. Yue F. Chen H. Hao Y. (2022). Lianhuaqingwen alleviates p53-mediated apoptosis in alveolar epithelial cells to prevent LPS-Induced ALI. J. Pharm. Pharmacol. 74 (8), 1117–1124. 10.1093/jpp/rgac035 35640566

[B54] Yin M. Marrone L. Peace C. G. O'Neill L. A. J. (2023). NLRP3, the inflammasome and COVID-19 infection. QJM 116 (7), 502–507. 10.1093/qjmed/hcad011 36661317 PMC10382191

[B55] Yu L. Che R. Zhang W. Xu J. Lian W. He J. (2023). Cornuside, by regulating the AGEs-RAGE-IκBα-ERK1/2 signaling pathway, ameliorates cognitive impairment associated with brain aging. Phytother. Res. 37 (6), 2419–2436. 10.1002/ptr.7765 36781177

[B56] Zhang X.-P. Zhang W.-T. Qiu Y. Ju M.-J. Yang C. Tu G.-W. (2020). Cyclic helix B peptide alleviates sepsis-induced acute lung injury by downregulating NLRP3 inflammasome activation in alveolar macrophages. Int. Immunopharmacol. 88, 106849. 10.1016/j.intimp.2020.106849 32795894

[B57] Zhang Y. Zhang J. Fu Z. (2022a). Molecular hydrogen is a potential protective agent in the management of acute lung injury. Mol. Med. 28 (1), 27. 10.1186/s10020-022-00455-y 35240982 PMC8892414

[B58] Zhang J. Pan W. Zhang Y. Tan M. Yin Y. Li Y. (2022b). Comprehensive overview of Nrf2-related epigenetic regulations involved in ischemia-reperfusion injury. Theranostics 12 (15), 6626–6645. 10.7150/thno.77243 36185600 PMC9516229

[B59] Zhang M. Lan H. Jiang M. Yang M. Chen H. Peng S. (2025). NLRP3 inflammasome mediates pyroptosis of alveolar macrophages to induce radiation lung injury. J. Hazard Mater 484, 136740. 10.1016/j.jhazmat.2024.136740 39642726

[B60] Zhao N. Di B. Xu L.-L. (2021). The NLRP3 inflammasome and COVID-19: activation, pathogenesis and therapeutic strategies. Cytokine Growth Factor Rev. 61 (3), 2–15. 10.1016/j.cytogfr.2021.06.002 34183243 PMC8233448

[B61] Zhou R. Yazdi A. S. Menu P. Tschopp J. (2011). A role for mitochondria in NLRP3 inflammasome activation. Nature 469 (7329), 221–225. 10.1038/nature09663 21124315

[B62] Zhou Y. Zhang Y. Wang H. Zhang X. Chen Y. Chen G. (2023). Microglial pyroptosis in hippocampus mediates sevolfurane-induced cognitive impairment in aged mice *via* ROS-NLRP3 inflammasome pathway. Int. Immunopharmacol. 116, 109725. 10.1016/j.intimp.2023.109725 36764275

[B63] Zhu X. Zhang H.-W. Chen H.-N. Deng X.-J. Tu Y.-X. Jackson A. O. (2019). Perivascular adipose tissue dysfunction aggravates adventitial remodeling in obese mini pigs *via* NLRP3 inflammasome/IL-1 signaling pathway. Acta Pharmacol. Sin. 40 (1), 46–54. 10.1038/s41401-018-0068-9 30002491 PMC6318288

