# Supplementary material for: Cornuside mitigates acute lung injury through suppression of NLRP3 inflammasome-mediated pyroptosis and activation of the Keap1-Nrf2 antioxidant response
Source: Front Pharmacol. 2025 Oct 8;16:1682523. doi: 10.3389/fphar.2025.1682523 (PMC12541256; doi:10.3389/fphar.2025.1682523)
Supplement: Supplementary file 2 [file Supplementaryfile1.docx]

Supplementary materials for “Cornuside Mitigates Acute Lung Injury Through Suppression of NLRP3 Inflammasome-Mediated Pyroptosis and Activation of the Keap1-Nrf2 Antioxidant Response”

**Supplementary Fig. 1 Schematic of the cell experimental strategy.** Diagram outlining the experimental design for in *vitro* cell studies.

**Supplementary Fig.2 Effects of CNS on behavioral test** **before and after ALI in mice** **a)** Vertical activity scores from the open field test were measured before and after ALI induction. Data are presented as mean ± SD (ns, not significant).

**Supplementary Fig. 3 CNS inhibits pyroptosis in *vitro*. a, b)** Cytotoxicity assessment of J774A.1 cells after 12 and 24 hours of CNS treatment (n = 3 per group). **c, d)** Fluorescence images of BMDMs stained with propidium iodide (PI) and Hoechst 33342; scale bar = 200 μm. Quantification of PI-positive cells was performed by counting six randomly selected fields (one field per well) (n = 5 per group). Data are presented as mean ± SD (*****P* < 0.0001).

**Supplementary Fig. 4 Effects of CNS on NLRP3 inflammasome. a, b)** Western blot analysis of NLRP3, pro-caspase-1, and ASC expression in the cell lysates of J774A.1 cells and BMDMs. Data are presented as mean ± SD (**P* < 0.05; ***P* < 0.01).
