# Supplementary figures and images for: Cornuside mitigates acute lung injury through suppression of NLRP3 inflammasome-mediated pyroptosis and activation of the Keap1-Nrf2 antioxidant response
Source: Front Pharmacol. 2025 Oct 8;16:1682523. doi: 10.3389/fphar.2025.1682523 (PMC12541256; doi:10.3389/fphar.2025.1682523)

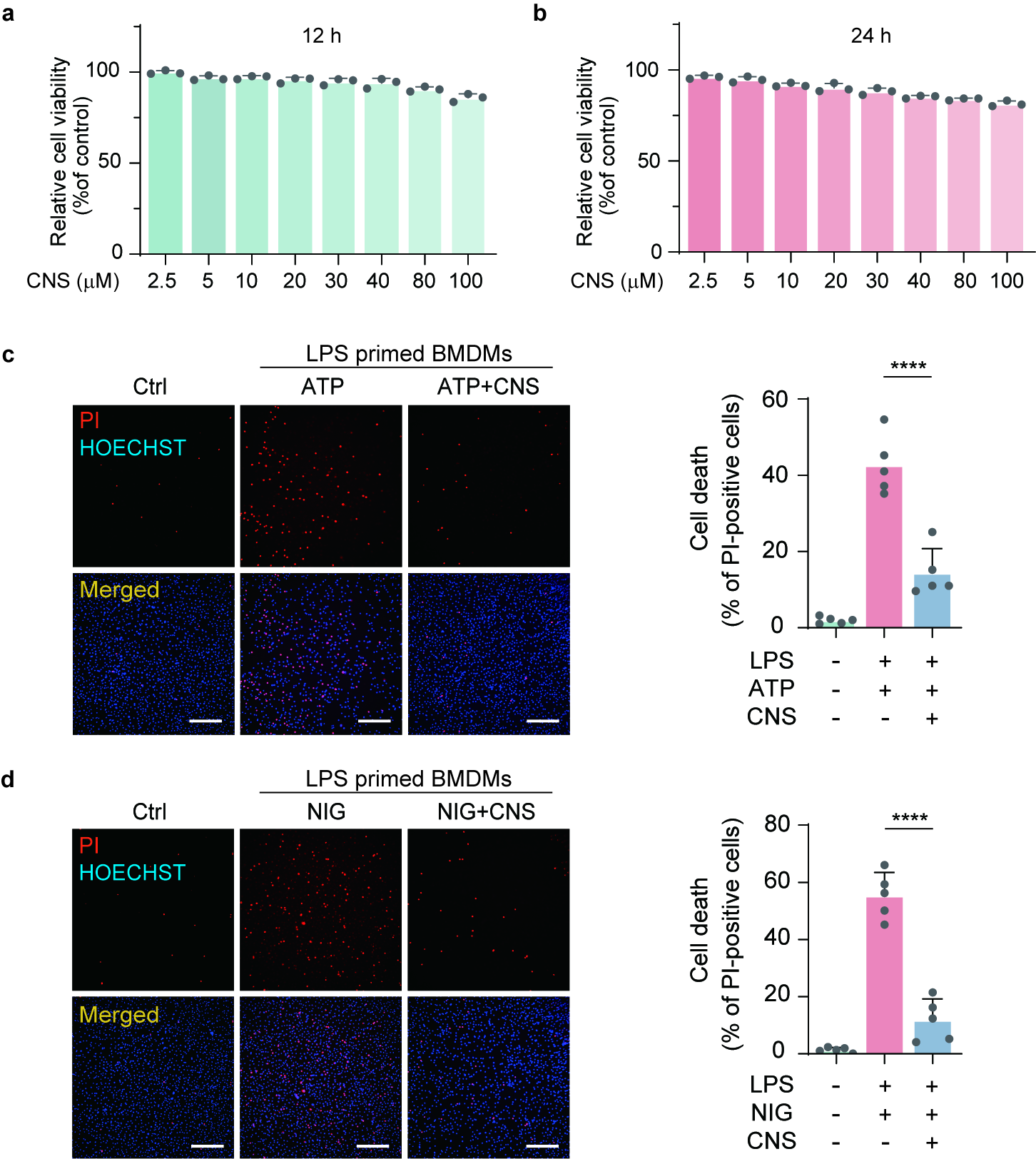

Supplement: Supplementary file 3 [file Image3.tif]

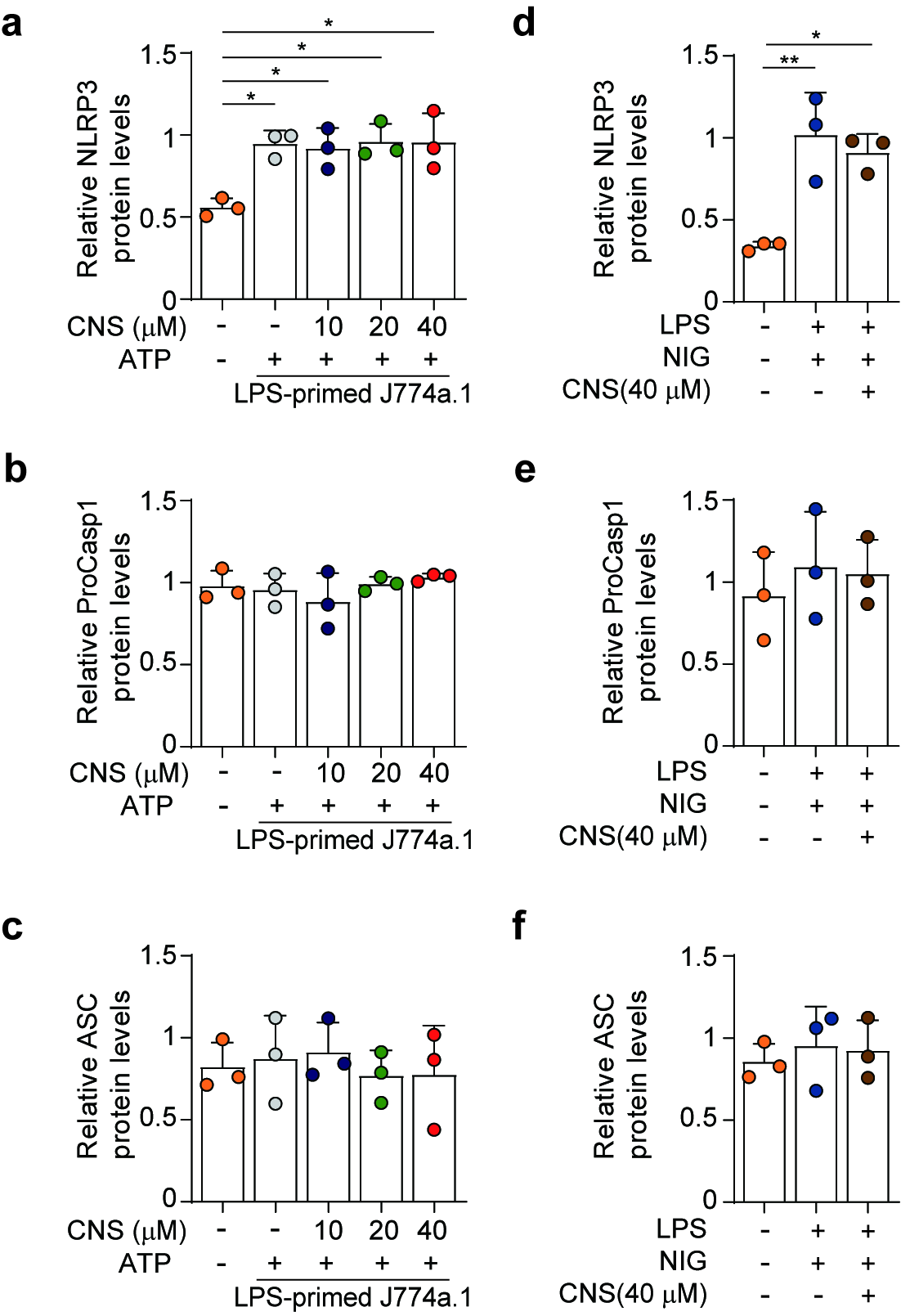

Supplement: Supplementary file 4 [file Image4.tif]

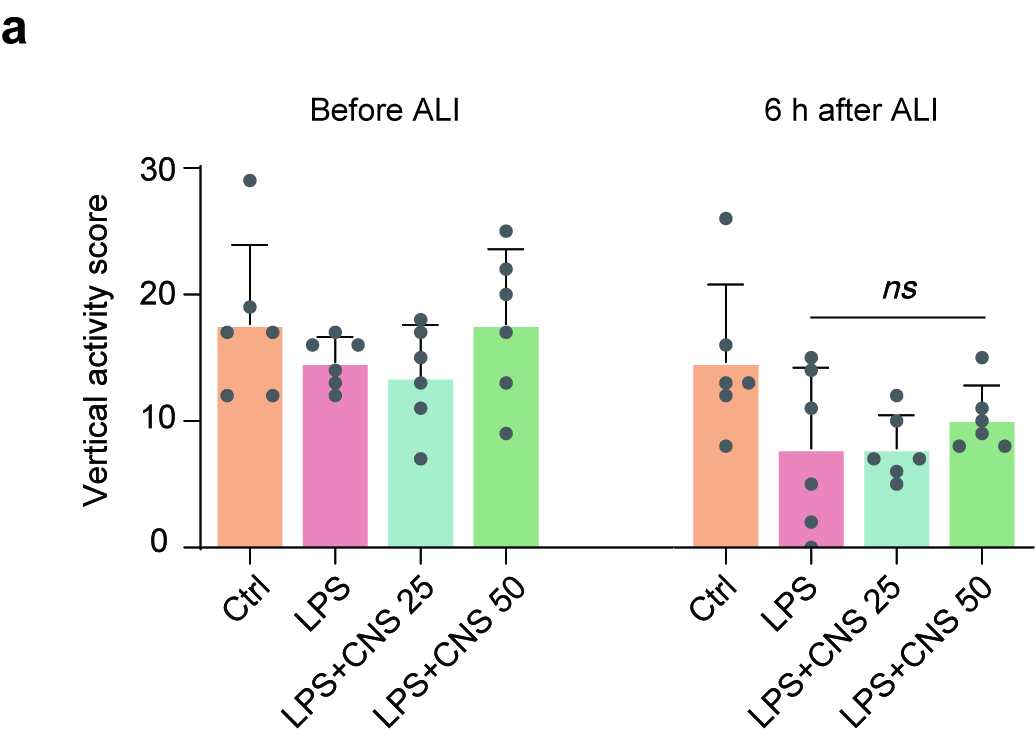

Supplement: Supplementary file 5 [file Image2.tif]

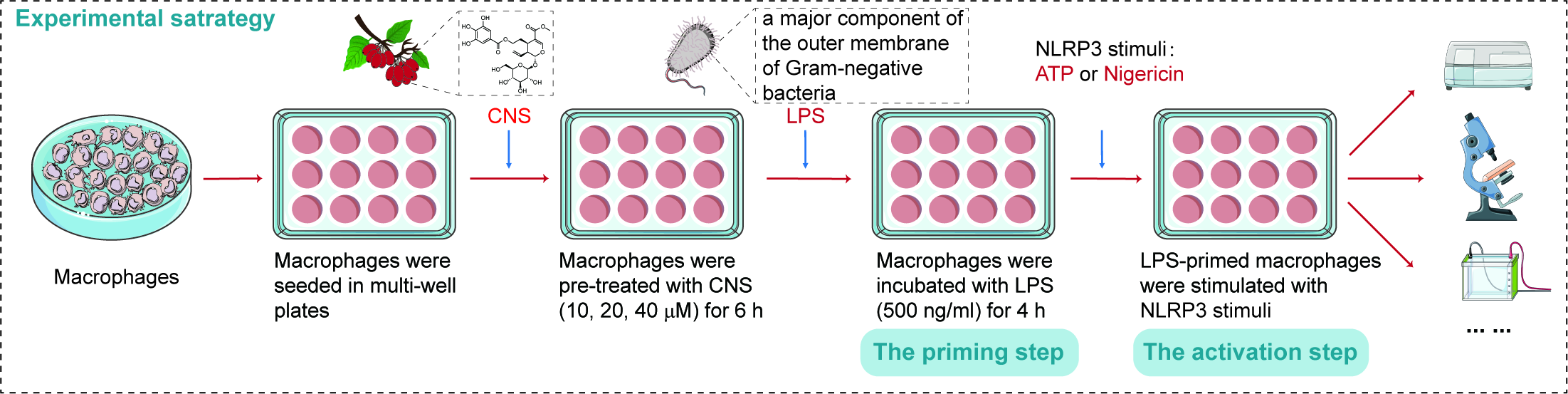

Supplement: Supplementary file 6 [file Image1.tif]
